# Suppression of colorectal tumorigenesis by recombinant *Bacteroides fragilis* enterotoxin-2 *in vivo*

**DOI:** 10.3748/wjg.v23.i4.603

**Published:** 2017-01-28

**Authors:** You Lv, Tao Ye, Hui-Peng Wang, Jia-Ying Zhao, Wen-Jie Chen, Xin Wang, Chen-Xia Shen, Yi-Bin Wu, Yuan-Kun Cai

**Affiliations:** You Lv, Tao Ye, Hui-Peng Wang, Jia-Ying Zhao, Wen-Jie Chen, Xin Wang, Chen-Xia Shen, Yi-Bin Wu, Yuan-Kun Cai, Department of General Surgery, The 5^th^ People’s Hospital of Shanghai, Fudan University, Shanghai 200240, China

**Keywords:** Colorectal neoplasms, *Bacteroides fragilis* toxin, Fragilysin, Recombinant proteins, Mice

## Abstract

**AIM:**

To evaluate the impact of recombinant *Bacteroides fragilis* enterotoxin-2 (BFT-2, or Fragilysin) on colorectal tumorigenesis in mice induced by azoxymethane/dextran sulfate sodium (AOM/DSS).

**METHODS:**

Recombinant proBFT-2 was expressed in *Escherichia coli* strain Rosetta (DE3) and BFT-2 was obtained and tested for its biological activity *via* colorectal adenocarcinoma cell strains SW-480. Seventy C57BL/6J mice were randomly divided into a blank (BC; *n* = 10), model (AD; *n* = 20), model + low-dose toxin (ADLT; *n* = 20, 10 μg), and a model + high-dose toxin (ADHT; *n* = 20, 20 μg) group. Mice weight, tumor formation and pathology were analyzed. Immunohistochemistry determined Ki-67 and Caspase-3 expression in normal and tumor tissues of colorectal mucosa.

**RESULTS:**

Recombinant BFT-2 was successfully obtained, along with its biological activity. The most obvious weight loss occurred in the AD group compared with the ADLT group (21.82 ± 0.68 *vs* 23.23 ± 0.91, *P* < 0.05) and the ADHT group (21.82 ± 0.68 *vs* 23.57 ± 1.06, *P* < 0.05). More tumors were found in the AD group than in the ADLT and ADHT groups (19.75 ± 3.30 *vs* 6.50 ± 1.73, *P* < 0.05; 19.75 ± 3.30 *vs* 6.00 ± 2.16, *P* < 0.05). Pathology showed that 12 mice had adenocarcinoma and 6 cases had adenoma in the AD group. Five mice had adenocarcinoma and 15 had adenoma in the ADLT group. Four mice had adenocarcinoma and 16 had adenoma in the ADHT group. The incidence of colorectal adenocarcinoma in both the ADHT group and the ADHT group was reduced compared to that in the AD group (*P* < 0.05, *P* < 0.05). The positive rate of Ki-67 in the ADLT group and the ADHT group was 50% and 40%, respectively, both of which were lower than that found in the AD group (94.44%, *P* < 0.05, *P* < 0.05). Caspase-3 expression in the ADLT group and the ADHT group was 45% and 55%, both of which were higher than that found in the BC group (16.67%, *P* < 0.05, *P* < 0.05).

**CONCLUSION:**

Oral administration with lower-dose biologically active recombinant BFT-2 inhibited colorectal tumorigenesis in mice.

**Core tip:** *Bacteroides fragilis* enterotoxin-2 (BFT-2) has been considered to promote the development of colorectal cancer (CRC). In this study, we obtained the biologically active recombinant BFT-2 *in vitro* and found that lower-dose of biologically active BFT-2 could inhibit colorectal tumor formation *via* the route of intra-gastric administration in a mice model of CRC, which manifested to inhibit cell proliferation and promoted apoptosis. The specific mechanism is still unknown but the findings provide some insights into prevention and treatment of CRC as an intestinal mucosal vaccine.

## INTRODUCTION

Colorectal cancer (CRC) is one of the most common malignant tumors of the digestive tract[1]. The relationship between intestinal flora and CRC is an intense area of recent research[2,3]. Preliminary observations showed that the proportion of *Bacteroides* in patients with CRC increased and was accompanied by reduced diversity[4].

*Bacteroides fragilis* (*B. fragilis*) is an obligate anaerobe that colonizes the lower digestive tract of humans. Enterotoxigenic *B. fragilis* (ETBF) is a subtype of *B. fragilis*, which can specifically secrete an extracellular 20 kDa zinc-dependent metalloproteinase referred to as *B. fragilis* toxin (BFT, or Fragilysin)[5,6]. BFT can damage the tight junction of the intestines, increase intestinal permeability, and provoke diarrhea[7]. Recent studies have shown that BFT is the major virulence factor of ETBF, and plays an important role in the occurrence and development of CRC[8-10].

The current study aimed to obtain BFT-2 *via* genetic engineering and to evaluate the impact of BFT-2 on the formation of colorectal tumor by utilizing an AOM/DSS-induced mouse model of CRC.

## MATERIALS AND METHODS

### Materials

**Plasmid and strain:** Recombinant plasmid pET-32a containing the target gene[11], pro-*B. fragilis* enterotoxin-2 without the signal peptide nucleotide sequence (proBFT-2), and positive clones of *Escherichia coli* (*E. coli*) strain Rosetta (DE3) were provided by Shanghai Biotechnology Corporation (GenBank ID: AB026626).

**Cell-line:** The human colorectal adenocarcinoma cell-line SW-480 was purchased from Shanghai Cell Bank of the Chinese Academy of Science.

**Animal experiments:** Seventy SPF certified C57BL/6J mice with a 17-g body weight, aged 5 wk were purchased and fed in the Animal Center of the Institute of Biomedical Laboratory of the East China Normal University. The production license was SCXK (Shanghai) 2011-0031, and the usage license was SYXK (Shanghai) 2010-0094. The animal experiment was approved by the 1990 Animal Ethics Committee of the East China Normal University, and the controlled environmental factors for experimentation strictly followed the relevant regulations of the national standard GB14925-2010 of the experimental animals that were released and implemented by the National Administration of Quality Supervision and Quarantine. All animals were permitted free access to food and water.

**Reagents:** Ni-NTA agarose gels were purchased from GE (United States). DMEM medium was purchased from Invitrogen (United States). IPTG was purchased from Sigma (United States). TEV protease was provided by Bioengineering (Shanghai, China). Azomethane (AOM) and dextran sulfate sodium (DSS) that was used to induce a state of CRC in the experimental mice were purchased from Sigma and MP (United States), respectively. Ki-67 and Caspase-3 antibodies were purchased from Abcam (United Kingdom).

### Methods

**Expression and purification of recombinant protein proBFT-2:** The nucleotide sequence coding for proBFT-2 without the signal peptide nucleotide sequence as the target gene was cloned to construct a recombinant plasmid pET-32a to produce the positive transformed clone strain DE3. Then, the positive transformed clone strains were cultured on a small scale, induced by IPTG, screened for the optimal condition of IPTG induction concerning concentration, temperature and time, and the SDS-PAGE method was utilized to select the best protein expression-inducing conditions. Then, the positive clones of DE3 with optimized inducing conditions were induced on a large scale. After IPTG induction of expression and cell sonication, the culture was centrifuged and the supernatants are applied to Ni-agarose affinity chromatography. The eluate was collected and tested by SDS-PAGE to detect recombinant proBFT-2 expression.

**Analysis of the purified proBFT-2:** ProBFT-2 was mixed with the TEV enzyme to remove the His-tag, and then an enzyme-digested protein solution was purified by nickel agarose affinity column chromatography. Finally, SDS-PAGE was used to detect the sample solution and elution before and after enzyme digestion.

**ProBFT-2 digestion and hydrolytic release of BFT-2:** In this procedure, 10 μg/mL trypsin was incubated with proBFT-2 solution at 37 °C for 1 h, leading to release of BFT-2 by proBFT-2 protein hydrolysis, following which it was purified by nickel column affinity chromatography, and the eluted fractions were collected. After the elution was dialyzed and purified, SDS-PAGE was performed. Finally, the elution was preserved at -80 °C.

**Detection of BFT-2 biological activity:** During the log phase of growth, 5 × 10^5^ SW-480 cells were seeded into a 24-well plate with a total volume of 50 μL of DMEM per well. The cells were incubated with 5% CO_2_ at 37 °C for 2 d. Then, the medium was replaced with FBS-free DMEM, and 2 μg/mL BFT-2 was added to a final volume of 1 mL. After that, morphological changes were observed by optical microscopy at 20, 40, 60, 90 and 120 min, respectively[12].

**Grouping for the animal experiment:** After 1 wk of adaptive observation, the mice were divided into four groups: the blank control group (BC), the AOM/DSS group (AD), the AOM/DSS + low-dose toxin group (ADLT), and the AOM/DSS + high-dose toxin group (ADHT). Mice in the BC group drank and ate freely, while mice in the AD group were intraperitoneally injected with AOM (0.2 mg/kg) on the first day of the first week. From the second week onwards, the mice in the AD group were given 2% DSS-free drinking water, which lasted 1 wk. At the third week, the water provided for mice in the AD group was switched to normal drinking water. In addition, a 3-wk period was considered to be one cycle and the process lasted for three cycles in total. Based on the AD group, mice in the ADLT group and the ADHT group received intra-gastric administration of 10 μg and 20 μg BFT-2 on the first day of DSS drinking, and mice in the other groups were given an equal volume of saline as the control[13,14].

**Observation of the general condition of the animals:** The general conditions of the mice were observed during the experiment, including hair color, mental status, activities, feeding, defecation, nutritional status, presence or absence of anal bleeding, and rectal prolapse. Body weight of the mice was weighed and recoded once a week. At the end of wk 14, the mice were sacrificed by cervical dislocation after fasting for 12 h.

**Status of tumor formation:** The abdominal cavity was fully exposed after the mice were sacrificed, and the large intestine was dissociated and completely removed. Along the longitudinal axis, the intestinal tract was split and flattened. Then, PBS buffer was used to wash the intestinal tract. Finally, the number, location and the size of the tumors, and the length of the large intestine were recorded.

**Histopathological examination:** Normal tissue of the colorectal mucosa and tumor tissues were selected and fixed in 4% paraformaldehyde solution. After fixing, the following steps were performed and included dehydration, transparency, embedding with paraffin, and HE staining. Finally, the lesion level of the induced CRC was evaluated for each group.

**Immunohistochemical examination of normal colorectal mucosal tissues and tumor tissues:** The expressions of Ki-67 and Caspase-3 in the specimens of normal colorectal mucosal tissues and tumor tissues were measured by SP staining. Ki-67 is expressed in the nucleus, and Caspase-3 is expressed in the cytoplasm. Semi-quantitative scoring was used. Based on the staining intensity, the specimen was classified according to the following levels: negative staining was considered as 0 points, weakly positive staining (+, pale yellow) as 1 point, moderately positive staining (++, brown) as 2 points and strongly positive staining (+++, tan) as 3 points. Furthermore, the specimen was scored based on the proportion of the number of positive cells. According to the range of positive staining, 5% was considered as 0 points, 5%-25% as 1 point, 26%-50% as 2 points, and 51%-75% as 3 points; greater than 75% was considered as 4 points. Statistics were performed for positive immunoreactivity when the product of the staining intensity and the proportion of the range of positive observations was greater than or equal to 2.

### Statistical analysis

SPSS version 20.0 statistical software was used to perform statistical analysis for the data. Continuous data that showed normal distribution was represented as mean ± SD, and the independent sample Student’s *t*-test was used to perform comparisons of the data. For comparisons of categorical data of two independent groups, the χ^2^ test was used. *P* < 0.05 was considered a statistically significant difference.

## RESULTS

### Expression and purification of recombinant protein BFT-2

**Figure 1:**
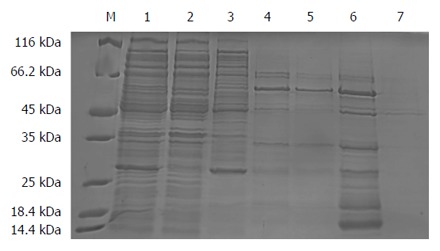
SDS-PAGE (10%) analysis for nickel agarose affinity chromatography purification of fusion protein. A new protein band with a molecular weight of approximately 55 kDa appeared in the 500 mmol/L imidazole elution fractions. M: Protein marker; 1: Sample; 2: Outflow; 3: 20 mmol/L Imidazole elution fractions; 4, 5: 50 mmol/L Imidazole elution fractions; 6, 7: 500 mmol/L Imidazole elution fractions.

**Figure 2:**
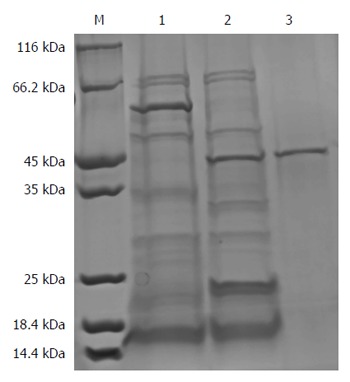
SDS-PAGE (10%) analysis for nickel agarose affinity chromatography purification of fusion protein after TEV digestion. A protein band of about 42 kDa appeared after the new protein was cut by TEV enzyme. M: Protein marker: 1: Before TEV digestion; 2: After TEV digestion (sample); 3: Outflow.

**Figure 3:**
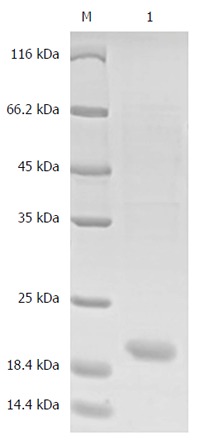
SDS-PAGE (10%) analysis of proBFT-2 hydrolysis using trypsin. A new protein band with a molecular of 20 kDa appeared after the protein was digested with trypsin. M: Protein marker; 10 μg/mL trypsin hydrolyzes proBFT-2 at 37 °C for 1 h.

The result showed that a new protein band with a molecular weight of approximately 55 kDa appeared in the 500 mmol/L imidazole elution fractions, which was larger than that of the target protein proBFT-2 (42 kDa) (Figure 1). After the new protein was cut by TEV enzyme, a protein band of about 42 kDa appeared, which was in line with the molecular weight of proBFT-2 (Figure 2). After the protein was digested with trypsin, re-detection found a new protein band with a molecular weight of 20 kDa, which was consistent with the molecular weight of BFT-2 (Figure 3).

### Detection of BFT-2 biological activity

**Figure 4:**
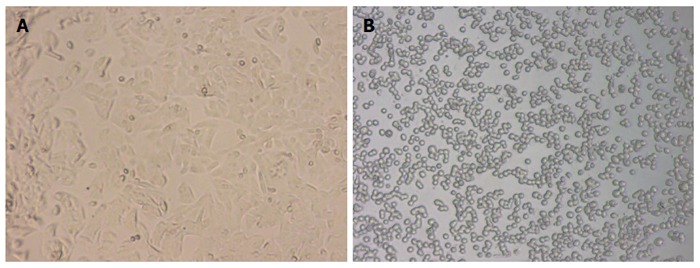
Before BFT-2 treatment (A) and after BFT-2 treatment (B). SW-480, which grows adherently in a fusiform shape under a normal condition, showed cell rounding and separating from each other after treatment with BFT-2.

SW-480 is a human colorectal adenocarcinoma cell line, which grows adherently in a fusiform shape under a normal condition. After treatment with 2 μg/mL BFT-2 at 37 °C for 1 h, the SW-480 cells were observed by an optical microscope and found to show obvious morphological changes, such as cell rounding and separating from each other. This demonstrated that BFT-2 had biological activity[7,15] (Figure 4).

### General status of the animals

**Figure 5:**
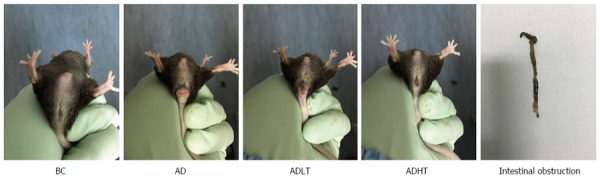
General status of mice in each group. BC: Normal; AD: Rectal prolapse; ADLT: Tumor prolapse; ADHT: Bloody stools, intestinal obstruction.

At the initial 7 wk, the mice in each group had normal diet and defecation, and no red and swollen anus was found. Moreover, the mice were reactive to irritants. At 8 wk, the mice in the AD group began to present with messy and dull hair, lags in response, curled and lack of exercise and reduced diet, and some mice had loose stools accompanied by blood or black mucous secretions, while rectal prolapse occurred in 1 mouse. At 10 wk, the mice in the ADLT group and the ADHT group showed reduced appetite, fatigue, lack of exercise, and loose stools. At 14 wk, all the mice in the AD group had bloody stools accompanied by blood mucous secretions that was loose; in addition, rectal prolapse occurred in these mice, and 2 mice died. However, 8 mice in the ADLT group and 5 mice in the ADHT group showed rectal prolapse; in addition, bloody secretion and black stool appeared, but no death was found (Figure 5).

**Figure 6:**
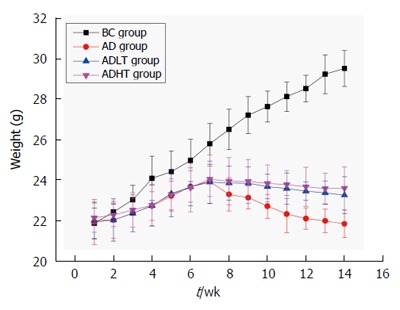
Alteration of body weight of mice of each group. Body weight of the mice in the BC group showed a steady growth. Significant decline appeared in the AD group since 8 wk, compared with the ADLT and ADHT groups. At 14 wk, the body weight of mice in the BC group was 29.48 ± 0.88 g, in the AD group was 21.82 ± 0.68 g, in the ADLT group was 23.23 ± 0.91 g, and in the ADHT group was 23.57 ± 1.06 g. AD *vs* ADLT, *P* = 0.006; AD *vs* ADHT, *P* = 0.002; ADLT *vs* ADHT, *P* = 0.632.

Body weight of the mice was not statistically significant when they were enrolled in the experiment. Body weight of the mice in the BC group showed a steady growth. At 8 wk, body weight of the mice in the AD group had decreased slowly. Moreover, it decreased slower in the ADLT group and the ADHT group as compared with the AD group. At 10 wk, body weight of the mice between the AD group and either the ADLT group or the ADHT group was statistically significant. At 14 wk, body weight of the mice in the BC group was 29.48 ± 0.88 g, in the AD group was 21.82 ± 0.68 g, in the ADLT group was 23.23 ± 0.91 g, and in the ADHT group was 23.57 ± 1.06 g. The differences of the changes of average body mass of the mice in the BC group were all statistically significant with those among the AD group, the ADLT group and the ADHT group (*P* =0.0001, *P =* 0.0001 and *P =* 0.0001, respectively). There was a significant difference in the alteration of the average body weight of the mice in the AD group when compared to the ADLT group and the ADHT group (*P =* 0.006 and *P =* 0.002, respectively), but there was no statistical difference in the alteration of the average body weight of the mice between the ADLT group and the ADHT group (*P =* 0.632) (Figure 6).

### Status of tumor formation

**Figure 7:**
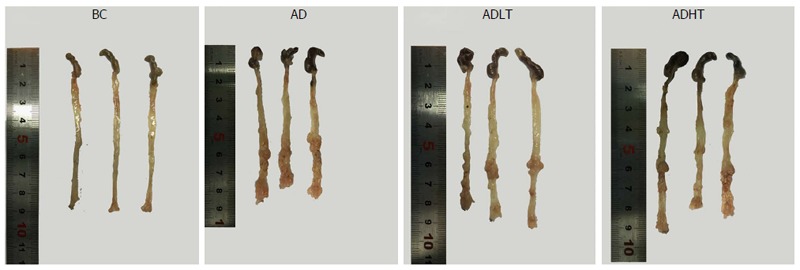
Comparison of the samples *in vitro* of the large intestine of mice from each group. All the mice in the BC group showed no occurrence of the formation of tumors. In the AD group, the ADLT group and the ADHT group, multiple tumors of different sizes were found on the mucosal surface and located in the distal site of the large intestine and the anal canal, which were accompanied by different degrees of colon shortening.

All the mice in the BC group showed no occurrence of formation of tumors, and the length of large intestine was 8.90 ± 0.10 cm. In the AD group, the ADLT group and the ADHT group, multiple tumors with different sizes were found on the mucosal surface, and these tumors were placed into the intestine tract. In addition, most of these tumors were located in the distal site of the large intestine and in the anal canal, which were accompanied by different degrees of colon shortening. The length of the large intestine was 8.10 ± 0.4 cm, 8.27 ± 0.31 cm and 8.20 ± 0.1 cm in the AD group, ADLT group and ADHT group, respectively. Furthermore, the number of formed tumors was 19.75 ± 3.30, 6.50 ± 1.73 and 6.00 ± 2.16 in the AD group, ADLT group and ADHT group, respectively. In addition, the average diameter was 1.72 ± 0.40 mm, 1.15 ± 0.41 mm and 1.07 ± 0.40 mm in the AD group, ADLT group and ADHT group, respectively. The length of the large intestine was shorter in the AD group, the ADLT group and the ADHT group, compared with that in the BC group (*P* = 0.027, *P* = 0.028 and *P =* 0.001, respectively), and there was no statistical difference among the AD group, the ADLT group and the ADHT group (*P =* 0.793). The number of tumors in the AD group was significantly higher than that in the ADLT group and the ADHT group, and the difference was statistically significant (*P =* 0.0001 and *P =* 0.0001, respectively), but there was no statistical difference between the ADLT group and the ADHT group. The average diameter of tumors in the AD group was larger than that in the ADLT group and the ADHT group (*P =* 0.006 and *P =* 0.002, respectively), but there was no difference between the ADLT group and the ADHT group (*P =* 0.74; Figure 7, Table 1).

**Table 1:** Comparison of the average lengths of the large intestine, the numbers of tumors and the diameters of tumors of mice from each group

	**BC group**	**AD group**	**ADLT group**	**ADHT group**
Length of the large intestine, cm	8.90 ± 0.10	8.10 ± 0.40^1^	8.27 ± 0.31^1^	8.20 ± 0.10^1^
Number of tumors	-	19.75 ± 3.30	6.50 ± 1.73^2^	6.00 ± 2.16^2^
Diameter of tumors, mm	-	1.72 ± 0.40	1.15 ± 0.41^3^	1.07 ± 0.40^3^

1 Represents a statistical significance in the length of the large intestine when compared between the BC group and the AD group or the ADLT group or the ADHT group (*P* < 0.05, respectively);

2 Represents that the number of tumors in the AD group was statistically different from those in the ADLT group and the ADHT group (*P* < 0.05, respectively);

3 Represents that the average diameter of tumors in the AD group was statistically different from those in the ADLT group and the ADHT group (*P* < 0.05, respectively).

### Histopathologic changes

**Figure 8:**
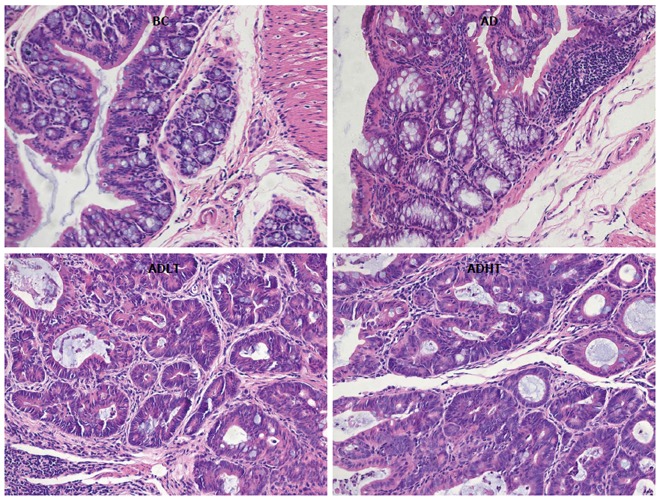
Comparison of the tissue of the large intestine of mice from each group. HE staining, magnification × 200.

There were 12 cases of adenocarcinoma and 6 cases of adenoma in the AD group, 5 cases of adenocarcinoma and 15 cases of adenoma in the ADLT group, and 4 cases of adenocarcinoma and 16 cases of adenoma in the ADHT group. The incidence of CRC in both the ADHT group and the ADHT group was reduced compared to that in the AD group (*P =* 0.0001 and *P =* 0.0001, respectively), and there was no significant difference between the ADLT group and the ADHT group (*P >* 0.05). The CRC induced in the experiment were almost exclusively adenocarcinomas, which were well-differentiated tubular adenocarcinomas in terms of histology (Figure 8).

### Expression of Ki-67 and Caspase-3 in the colon

**Figure 9:**
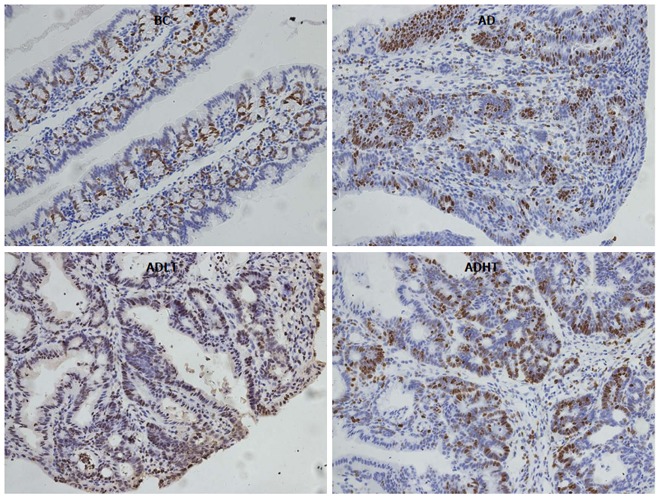
Expression of Ki-67 in the large intestine of mice from each group. SP staining, magnification × 200.

**Figure 10:**
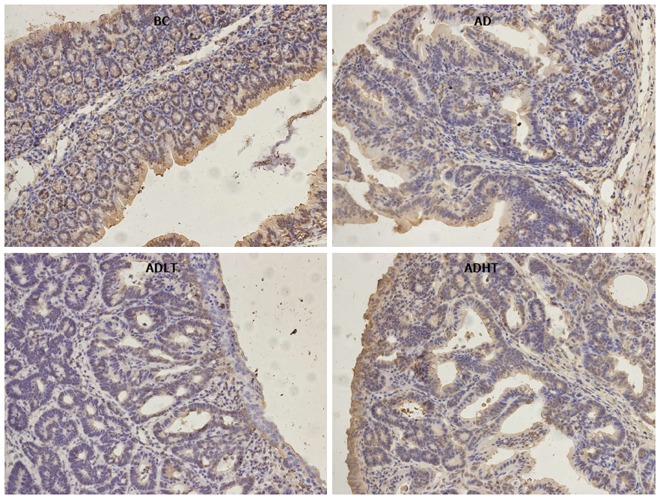
Expression of Caspase-3 in the large intestine of mice from each group. SP staining, magnification × 200.

When compared to the BC group, Ki-67 had higher positive expression in the AD group, the ADLT group and the ADHT group, with statistical difference (*P =* 0.0001, *P =* 0.0001 and *P =* 0.002, respectively). Furthermore, there was a statistical significance between the AD group and either the ADLT group or the ADHT group (*P =* 0.0001 and *P =* 0.0001, respectively), but no difference was found between the ADLT group and the ADHT group. There was statistically significant difference in Caspase-3 positive expression in the BC group when compared to the AD group, the ADLT group and the ADHT group (*P =* 0.0001, *P =* 0.0001 and *P =* 0.028, respectively). Moreover, the expression between the AD group and either the ADLT group or the ADHT group was statistically significant (*P =* 0.0001 and *P =* 0.0001, respectively), but there was no difference between the ADLT group and the ADHT group (Figures 9 and 10; Table 2).

**Table 2:** Expression of Ki-67 and Caspase-3 of mice from each group

**Group**	***n***	**Ki-67**		**Caspase-3**	
		**-**	**+**	**-**	**+**
BC	10	8 (80.00)	2 (20.00)	3 (30.00)	7 (70.00)
AD	18	1 (5.56)	17 (94.44)^1^	15 (83.33)	3 (16.67)^3^
ADLT	20	10 (50.00)	10 (50.00)^1,2^	11 (55.00)	9 (45.00)^3,4^
ADHT	20	12 (60.00)	8 (40.00)^1,2^	9 (45.00)	11 (55.00)^3,4^

^1,3^Represents that the expressions of Ki-67 and Caspase-3 in the BC group were statistically different when compared to that in the AD group or the ADLT group or the ADHT group (*P* < 0.05, respectively) and (*P* < 0.05, respectively); ^2,4^Represents that the expressions of Ki-67 and Caspase-3 in the AD group were statistically different with those in the ADLT group and the ADHT group (*P* < 0.05, respectively) and (*P* < 0.05, respectively).

## DISCUSSION

In recent years, the relationship between the intestinal flora and CRC has become a hot topic for research[16,17]. Previous studies have demonstrated that ETBF can promote the occurrence and development of CRC. Toprak et al[8] collected stool specimens of patients with CRC and from normal human subjects, and culture of those tissues and comparative analysis were performed. From those stool specimens, the identified ratio of *B. fragilis* was 77% and 68%, respectively (*P >* 0.05), and the positive ratio of BFT genes that were detected in the isolated strains of *B. fragilis* was 38% and 12%, respectively (*P* = 0.009). Therefore, it was preliminarily concluded that BFT was closely associated with the occurrence and development of CRC. Boleij et al[9] compared the presence of BFT genes in the colorectal mucosa between cases with CRC and cases in the blank control group, finding that the detection rates of the left colon were 85.7% and 53.1% (*P =* 0.03), and a detection rate of 91.7% and 55.5% in the right colon (*P =* 0.04). Furthermore, positive rates of BFT genes were 100% and 72.7% in advanced and early CRC (*P =* 0.09), respectively, suggesting that BFT was a risk factor for the formation of CRC. Wu et al[18] utilized APC^min^ mice [which are multiple intestinal neoplasia (Min) mice that are heterozygous for the adenomatous polyposis coli (*apc*) gene] to perform ETBF single colonization through intra-gastric administration. This experiment indicated that ETBF promotes proliferation of colon cancer through the IL-17/IL-23 pathway. However, it remains largely unknown with respect to how BFT plays a prominent role in human cancer.

The initial design of this study was to verify how BFT-2 promotes the occurrence and development of CRC and to identify the possible mechanisms. However, the result was completely unexpected, and derived for us a completely opposite conclusion. The study found that both the ADLT group and the ADHT group showed the inhibition of colon tumor formation when BFT-2 was used to treat AOM/DSS-induced mice. The performance of the disease burden in both the ADLT and the ADHT group were light and delayed as compared with those in the AD group, and body weight significantly decreased slowly (*P =* 0.006 and *P =* 0.002, respectively). At the end of the experiment, the average number of formed tumors in the ADLT and the ADHT groups was 6.50 ± 1.73 and 6.00 ± 2.16, respectively, which were reduced significantly when compared to the AD group (19.75 ± 3.30; *P =* 0.0001 and *P =* 0.0001 respectively). Meanwhile, pathological results showed that the occurrence rate of CRC was 25% in the ADLT group, while it was 20% in the ADHT group, which were reduced when compared with that found for the AD group (66.7%; *P =* 0.0001 and *P =* 0.0001, respectively).

In addition, Ki-67 had a high rate of positive expression in both the ADLT and the ADHT group, where it was 50% and 40%, respectively. The observations were statistically significant when compared with that found in the AD group (94.44%; *P* = 0.0001 and *P =* 0.0001, respectively). The expression of Caspase-3 was 45% and 55% in the ADLT and ADHT groups, respectively, which were also statistically significant as compared with that found in the BC group (16.67%; *P =* 0.0001 and *P =* 0.0001, respectively); this observation suggested that BFT-2 could inhibit the proliferation of cells and promote apoptosis.

ETBF can produce three different toxins, including BFT-1[19], BFT-2[20] and BFT-3[11], of which BFT-2 is the most active[21]. In this study, BFT-2 was shown to inhibit the formation of colorectal tumor in mice, and reflected a dual role of intestinal flora in the regulation of intestinal immune function. On one hand, some intestinal flora and their products as antigens can stimulate intestinal inflammation, causing DNA damage and further promoting the occurrence of cancer[2,22]. By contrast, they can regulate intestinal immunity, enhance the ability of intestinal immune cells to resist pathogens, improve pathogen tolerance, and down-regulate the release of inflammatory cytokines that carry with them the risk of promoting tumors; thus, the toxicants have the capacity to inhibit the occurrence of tumors[23,24]. This effect also explains why the progression of CRC has an intricate association with inflammatory signaling pathways of the intestinal system.

The conclusions drawn from our study were similar to those described in the study of Doulberis et al[14]. In that study, cholera toxin (CT) and a well-established mouse model of colon cancer was used in which tumor formation was initiated by a single dose of the genotoxic agent AOM, which subsequently promoted inflammation that was caused by the colitogenic DSS. Those authors found that a single and low non-pathogenic oral dose of CT administered at the beginning of each DSS treatment cycle down-regulated neutrophils and up-regulated regulatory T cells and IL-10 in the colonic mucosa.

CT-induced disruption of the tumor-promoting character of DSS-induced inflammation led to the reduction of AOM-initiated colonic polypoidogenesis. This result added value to the emerging notion that certain gastrointestinal tract bacteria or their products affect the immune system and render the microenvironment of preneoplastic lesions less favorable for promoting their evolution to cancer. Therefore, it is of great importance to develop specific bacterial antigens to regulate intestinal immunity, thus inhibiting intestinal tumorigenesis. These specific antigens may become safe and effective consolidation agents that can regulate intestinal immune function, which has the potential to control intestinal inflammatory in the future, and reduce the risk of relevant tumors[25].

In summary, this study successfully constructed, expressed and purified recombinant protein BFT-2 through genetic engineering techniques. BFT-2 blocked the formation of CRC with the performance that alleviated disease in a murine model, and reduced the number and size of the formed tumors in mice. This study further elaborated the mechanisms of BFT-2 in inhibiting the formation of CRC, and it also demonstrated that key signaling pathways can provide some insights into the mechanistic underpinnings for the prevention and treatment of CRC by employing an intestinal mucosal vaccine.

## ACKNOWLEDGMENTS

The authors would like to thank the Shanghai Health and Family Planning Commission, The 5^th^ People’s Hospital of Shanghai, and the Animal Center of the Institute of Biomedical Laboratory of the East China Normal University for their support.

## COMMENTS

### Background

The relationship between intestinal flora and colorectal cancer (CRC) is an intense area of recent research. Their preliminary observations showed that the proportion of *Bacteroides* in patients with CRC increased and was accompanied by reduced diversity. Enterotoxigenic *Bacteroides fragilis* (ETBF), a subtype of *Bacteroides fragilis* (*B. fragilis*) which subordinated to *Bacteroides*, can specifically secrete an extracellular 20-kDa zinc-dependent metalloproteinase referred to *B. fragilis* enterotoxin (BFT). As the major virulence factor of ETBF, BFT plays an important role in the occurrence and development of CRC, but the specific mechanism *in vivo* has not yet been elucidated.

### Research frontiers

Previous experiments have already proved that the *BFT* genes are overexpressed in CRC patients, compared to healthy volunteers, and that intra-gastric administration of ETBF to APC^min^ mice can accelerate the formation of CRC through the IL-17/IL-23 pathway.

### Innovations and breakthroughs

This is the first study evaluating the impact of recombinant BFT-2 on colorectal tumorigenesis in mice induced by azoxymethane/dextran sulfate sodium (AOM/DSS). The results showed that BFT-2 could inhibit colorectal tumor formation in mice, mainly reflected by alleviated disease manifestations, reduced colorectal tumor numbers and size, and inhibited formation of colorectal adenocarcinoma. It was also suggested that BFT-2 led to inhibited cell proliferation and promoted apoptosis. As there is no doubt for the outcomes.

### Applications

The authors speculated that oral administration with lower-dose BFT-2 could regulate intestinal immune function, enhance the intestinal disease tolerance and inhibit tumorigenesis similar to intestinal mucosal vaccine. The results reflect the dual role of intestinal flora and its metabolites in the occurrence and development of CRC, but its specific mechanism still needs further exploration.

### Terminology

BFT is the major virulence factor of ETBF, which is a zinc-dependent metalloproteinase essentially. BFT is synthesized in intracellular bacteria and released to the extracellular environment with biological activity which can stimulate E-cadherin cleavage and destruction of tight junctions in intestinal epithelium. Three subtypes of BFT have been isolated, among which BFT-2 is the most active and better studied. A method for obtaining pure BFT-2 is extraction from culture medium using complicated procedures and yielding poor products.

### Peer-review

The authors evaluated the impact caused by BFT-2 after induced oncogenesis of colorectal tumors with AOM/DSS in mice. Though the initial purpose of the study was to verify if BFT-2 was able to further stimulate cancer development, the authors found that BFT could actually inhibit tumor formation. It was also suggested that BFT-2 led to inhibited cell proliferation and promoted apoptosis. Though it would be interesting to see a control group treated only with BFT, its absence should not affect the interpretation of obtained results. Overall, the study was well-designed and the findings appear to be robust.
